# Architecture of fibrin network inside thrombotic material obtained from the right atrium and pulmonary arteries: flow and location matter

**DOI:** 10.1007/s11239-012-0806-7

**Published:** 2012-10-19

**Authors:** Piotr Mazur, Robert Sobczyński, Michał Ząbczyk, Paulina Babiarczyk, Jerzy Sadowski, Anetta Undas

**Affiliations:** 1 Institute of Cardiology, Jagiellonian University Medical College, 80 Pradnicka St., 31-202 Kraków, Poland; 2 The John Paul II Hospital, Kraków, Poland

**Keywords:** Pulmonary embolism, Right atrium, Thrombus architecture, Fibrin

## Abstract

Pulmonary embolectomy is a treatment option in selected patients with high-risk pulmonary embolism (PE). Efficiency of thrombus degradation in PE largely depends on the architecture of its fibrin network, however little is known about its determinants. We present the case of a 56-year-old woman with high-risk PE and proximal deep-vein thrombosis, whose thrombotic material removed during embolectomy from the right atrium and pulmonary (lobar and segmental) arteries has been studied using scanning electron microscopy (SEM). SEM images showed that distally located thrombi are richer in densely-packed fibrin fibers and contain more white cells and less erythrocytes than the proximal ones and the atrial thrombus. Fibrin fibers alignment along the flow vector was observed in the thrombi removed from high-velocity flow pulmonary arteries, and not in the atrial thrombus. The content of denser fibrin network and platelet aggregates was increased in segmental thromboemboli. Our findings describe the relation between thrombus architecture and location, and might help to elucidate thrombus resistance to anticoagulant therapy in some PE patients.

## Introduction

Pulmonary embolism (PE) is associated with a substantial morbidity and mortality [1]. Right heart thrombi are usually found in hemodynamically compromised patients with acute PE and indicate worse prognosis [2]. In patients with high-risk PE and right heart dysfunction pulmonary embolectomy is a treatment option [3]. An autopsy-based study on pulmonary thromboembolus morphology suggested its structure to be layered, with layers of fibrin and platelets alternating with layers of erythrocytes [4].

Fibrin network, that represents a major component of venous thrombi, is lysed at a rate dependent on its architecture, with impaired lysis in case of dense clots [5]. Fibrin clots obtained from subjects with chronic thromboembolic pulmonary hypertension have been reported to be resistant to lysis [6]. In vitro experiments indicate that velocity of flow markedly affects fibrin clot structure [7]. With the increase of flow, more fibrin fibers are aligned along the flow vector, and less are randomly oriented [8].

Recently, we have shown that the structure of the pulmonary thrombus obtained during embolectomy is organized, fibrin-rich, and not layered [9]. To our knowledge, there is no ex vivo data on the architecture of right heart thrombi and pulmonary thromboemboli. Here we present the case of a 56-year-old woman with high-risk PE whose thrombotic material removed from right atrium and lobar and segmental pulmonary arteries has been studied using scanning electron microscopy (SEM).

## Case report

**Fig. 1:**
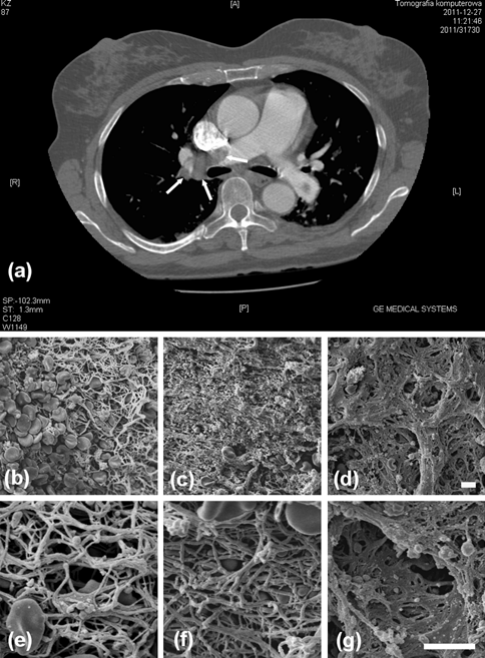
Computed tomography and scanning electron microscopic (SEM) images. Contrast-enhanced computed tomography showing a thrombus at the trifurcation of right pulmonary artery (**a**). Representative SEM images of the removed parts of the thrombus from the right atrium (**b** and **e**), lobar pulmonary artery (**c** and **f**) and segmental artery (**d** and **g**). **b**–**d** magnification, ×1,000; **e**–**g** magnification, ×3,500. *Scale bar* 5 μm

A 56-year-old woman (body mass index, 29.8 kg/m^2^), a former smoker, with a history of chronic venous insufficiency who suffered from recent-onset schizophrenia was admitted to our institution for PE after a 48 h treatment with unfractionated heparin in a regional hospital. Two months prior to admission she was hospitalized in a psychiatric ward and received no thromboprophylaxis. On admission, the patient presented with progressing dyspnea, cyanosis and chest discomfort with elevated D-dimer (>16,000 μg/l). Contrast-enhanced computed tomography revealed large emboli at the right pulmonary artery (PA) trifurcation and in the left PA (Fig. 1a). Extensive proximal DVT in both legs was observed. Transthoracic echocardiogram demonstrated right ventricular (RV) overload with RV systolic pressure exceeding 50 mmHg, and a right atrial mass originating in the inferior vena cava. Within 2 h she developed cardiogenic shock requiring inotropic support. Due to rapid clinical deterioration despite anticoagulant therapy, she underwent urgent embolectomy with extracorporeal circulation in deep hypothermia. Pulmonary trunk was opened, and fresh and organized thrombi were removed from right and left lobar and segmental branches. After opening of the right atrium, a 7 cm long fresh thrombus coming from the inferior vena cava was removed. The patient required prolonged ventilatory support and management of pulmonary hypertension during the post-operative period. Three-month follow-up was uneventful. She was treated with warfarin. Thrombophilia screening and family history of thrombosis were negative. Cancer as a cause of thrombosis was excluded.

The thromboemboli were evaluated using SEM, as previously described [10]. After washing, the thrombus was fixed with 2.5 % glutaraldehyde phosphate buffered saline solution. Specimens were dehydrated, gold coated, and photographed digitally with a JEOL JSM 5410 (JEOL, Tokyo, Japan).

The microscopic analysis revealed that the atrial thrombus was mainly composed of erythrocytes with few platelet aggregates, and some white cells (Fig. 1b, e). The fibrin network of the atrial thrombus was rather randomly arranged. In the thrombi removed from lobar (Fig. 1c, f) and segmental pulmonary arteries (Fig. 1d, g), a stepwise increase in relatively dense fibrin and platelet aggregates was observed. There was a noticeable decrease in the count of erythrocytes and increase in the count of white cells in the thrombotic material removed from lobar and segmental arteries, as compared with the atrial thrombus (Fig. 1). Thrombi removed from lobar and segmental arteries were characterized by fibrin fibers alignment along the longitudinal axis of the respective vessel, which was absent in the atrial thrombus.

## Discussion

To our knowledge, this study is the first to demonstrate the composition of right atrial and pulmonary thrombi removed during embolectomy in the PE patient. We observed that the fibrin content in pulmonary thrombus increases when the diameter of pulmonary arteries reduces, which is in contrast to the major impact of the time since symptom onset (“the clot’s ageing”), but not the vessel caliber, reported in acute myocardial infarction [11]. Moreover, the thrombus location appears to affect the architecture of fibrin network, since more tightly packed fibrin fibers resembling a solid mass were observed in thrombi from pulmonary arteries where the flow velocity is higher than in the right atrium. It is likely that the structural differences among the various thrombi, observed by us, result in part from hemodynamic conditions in the right atrium versus pulmonary arteries. Higher flow velocity in the pulmonary arteries might also contribute to lower number of erythrocytes, accompanied by increased fibrin content in the thrombi from those vessels. The increasing number of white cells within distal pulmonary thrombi indicates their early active role in their modulation and/or degradation in vivo. Interestingly, fibrin fiber alignment in distal pulmonary thrombi shown in this report confirms the recent in vitro findings, [7] clearly indicating a potent modulatory effect of flow on fibrin structure in vivo. It should also be highlighted that in pulmonary thrombi, platelets and fibrin are co-localized, and the presence of platelets determines the local formation of denser fibrin network via multiple mechanisms, including unfavorable impact of polyphosphate and platelet factor 4 on clot structure, reported in vitro [12]. Such architecture of the pulmonary thrombus might impede its dissolution despite administration of anticoagulants. This report provides an important in vivo evidence of the importance of platelet-related factors affecting fibrin clot architecture.

Mechanisms underlying such fibrin phenotype in pulmonary thromboemboli obtained from our PE patient remain to be elucidated. Most likely, transient factors, including those linked to hemodynamic abnormalities and massive thrombosis, determine thrombus properties to a greater extent than genetic factors in our case [5]. We cannot exclude that abnormal fibrin clot phenotype measurable prior to PE might contribute to unfavorable clinical course in this case and contribute to failure of heparin therapy. It might be speculated that unfavorable fibrin network characteristics with formation of compact fibrin structures in some patients with acute PE might explain, at least in part, heparin treatment failure.
